# Notch3 Modulates Cardiac Fibroblast Proliferation, Apoptosis, and Fibroblast to Myofibroblast Transition via Negative Regulation of the RhoA/ROCK/Hif1α Axis

**DOI:** 10.3389/fphys.2020.00669

**Published:** 2020-06-30

**Authors:** Jianli Shi, Peilin Xiao, Xiaoli Liu, Yunlin Chen, Yanping Xu, Jinqi Fan, Yuehui Yin

**Affiliations:** ^1^Department of Cardiology, The Second Affiliated Hospital of Chongqing Medical University, Chongqing, China; ^2^Department of Biomedical Engineering and Pediatrics, Emory University, Atlanta, GA, United States

**Keywords:** notch, cardiac fibroblast, cardiac fibrosis, myocardial infarction, extracellular matrix

## Abstract

Cardiac fibrosis is a common pathological process in multiple cardiovascular diseases, including myocardial infarction (MI). Abnormal cardiac fibroblast (CF) activity is a key event in cardiac fibrosis. Although the Notch signaling pathway has been reported to play a vital role in protection from cardiac fibrosis, the exact mechanisms underlying cardiac fibrosis and protection from it have not yet been elucidated. Similarly, Hif1α and the RhoA/ROCK signaling pathway have been shown to participate in cardiac fibrosis. The RhoA/ROCK signaling pathway has been reported to be an upstream pathway of Hif1α in several pathophysiological processes. In the present study, we aimed to determine the effects of notch3 on CF activity and its relationship with the RhoA/ROCK/Hif1α signaling pathway. Using *in vitro* experiments, we demonstrated that notch3 inhibited CF proliferation and fibroblast to myofibroblast transition (FMT) and promoted CF apoptosis. A knockdown of notch3 using siRNAs had the exact opposite effect. Next, we found that notch3 regulated CF activity by negative regulation of the RhoA/ROCK/Hif1α signaling pathway. Extending CF-based studies to an *in vivo* rat MI model, we showed that overexpression of notch3 by the Ad-N3ICD injection attenuated the increase of RhoA, ROCK1, ROCK2, and Hif1α levels following MI and further prevented MI-induced cardiac fibrosis. On the basis of these results, we conclude that notch3 is involved in the regulation of several aspects of CF activity, including proliferation, FMT, and apoptosis, by inhibiting the RhoA/ROCK/Hif1α signaling pathway. These findings are significant to further our understanding of the pathogenesis of cardiac fibrosis and to ultimately identify new therapeutic targets for cardiac fibrosis, potentially based on the RhoA/ROCK/Hif1α signaling pathway.

## Introduction

Heart failure (HF), secondary to ischemic or non-ischemic cardiomyopathy, is a serious health issue with high rates of associated morbidity and mortality. The progression of HF is in part caused by cardiac fibrosis, which is characterized by the deposition of extracellular matrix (ECM) and the activation of cardiac fibroblasts (CFs). Cardiac fibrosis is a common pathological process during the development of HF in multiple cardiovascular diseases (Li K. et al., 2019; Wang et al., 2019). Excessive cardiac fibrosis is furthermore a predictor of sudden cardiac death and overall mortality for cardiomyopathy (Gulati et al., 2013).

The myocardium consists of cardiomyocytes, CFs, and endothelial cells. CFs are predominantly involved in the maintenance of the ECM, which plays an important role in myocardial fibrosis (Prabhu and Frangogiannis, 2016). During myocardial fibrosis, CFs have a proliferative and migratory phenotype and exhibit enhanced secretion of ECM. This alteration in CF activity is a key regulatory event in cardiac fibrosis. Several proinflammatory and profibrotic factors are involved in the regulation of CF activity in cardiac fibrosis, such as NF-κB, bone morphogenetic protein, and TGF-β1 (Sun et al., 2013; Fix et al., 2019; Li A.Y. et al., 2019). These factors have been shown to be involved in similar pathological processes for several different etiologies (Harvey and Leinwand, 2011).

The Notch signaling pathway is a highly conserved signaling system involved in cellular differentiation, proliferation, apoptosis, and epithelial-to-mesenchymal transformation (EMT) (Hu and Phan, 2016; MacGrogan et al., 2018). Previous studies have demonstrated that the notch signaling pathway is able to protect the myocardium from ischemia (Zhou et al., 2019). However, the molecular mechanisms of notch3 in alleviating cardiac fibrosis are not fully elucidated, and further research is required.

Hif1 is part of the family of basic-helix-loop-helix/Per-ARNT-Sim (bHLH/PAS) DNA binding transcription factors (Greer et al., 2012) and is a major regulator of the hypoxic response. Hif1α is unstable and rapidly degraded by the ubiquitin–proteasome system. The role of Hif1α in cardiovascular diseases is controversial (Kido et al., 2005; Shyu et al., 2005a, b; Natarajan et al., 2008). In a review of previous studies, Xiong and Liu (2017) suggested that Hif1α may contribute to excessive ECM deposition and vascular remodeling and thus constitutes a vital therapeutic target for fibrotic diseases. The majority of previous studies investigating Hif1α was looking at the hypoxia pathway, but Hif1α is involved in a variety of other pathways. For example, angiotensin II (Ang II) has been shown to increase Hif1α levels in vascular smooth muscle cells independent of the oxygen environment (Richard et al., 2000). Furthermore, the RhoA/ROCK signaling pathway has been found to act as an upstream pathway of Hif1α in several pathophysiological processes, constituting a molecular switch in regulating cellular adherence, proliferation, and apoptosis (Sarrabayrouse et al., 2007; Jing et al., 2015; Rao et al., 2017). There is mounting evidence suggesting that RhoA/ROCK participates in the regulation of fibrosis by interacting with other signaling pathways or regulators (Bei et al., 2016; Tang et al., 2018; Zhou et al., 2018; Lai et al., 2019). However, no studies have so far clearly demonstrated the mechanisms underlying the regulation of CF activity via interaction of the notch signaling pathway with Hif1α and RhoA/ROCK. To explore these signaling events in cardiac fibrosis, we investigated the effects of Hif1α–RhoA/ROCK interaction on the modulation of notch-dependent fibrotic events under normoxia. Our results showed that notch3 regulated CF activity *in vitro* and cardiac fibrosis *in vivo*. Besides, we confirmed the involvement of the RhoA/ROCK/Hif1α signaling pathway in these processes.

## Materials and Methods

### Animal Care and Procedures

Six- to eight-week-old male Sprague–Dawley rats (weighing 250 ± 20 g) were obtained from the Animal Research Center of Chongqing Medical University (Chongqing, China) and housed in a temperature-controlled environment on a 12-h/12-h light/dark cycle. All experimental procedures were approved by the Institutional Ethics Committee of Chongqing Medical University. The rats were anesthetized using sodium pentobarbital (60 mg/kg, i.p.), and thoracotomy was performed. We injected N3ICD (notch3 intracellular domain)-expressing adenovirus (Ad-N3ICD) and GFP-expressing adenovirus (Ad-GFP) [purchased from Genechem (Shanghai, China)] into the free anterior wall of the left ventricle at five different sites (2 × 10^9^ pfu/ml, 5 μl per injection). Two days later, we introduced a myocardial infarction (MI) model as previously published (Qian et al., 2019). Briefly, ligation was accomplished at the proximal third of the left anterior descending (LAD) artery, and then the anterior wall of the left ventricle turned pale. Two months after thoracotomy, the rats were sacrificed, and the ventricular myocardium from the ischemic or region bordering the scar was harvested for further experiments.

### Echocardiography

Transthoracic echocardiography was performed using Toshiba Aplio 500 ultrasound system equipped with a linear transducer probe (PLT-1204BT) at 2 months after thoracotomy. Two-dimensional and M-mode echocardiography was obtained both in parasternal short- and long-axis views. Left ventricular end-diastolic diameter (LVEDD) and calculated left ventricular ejection fraction (LVEF) were acquired on heart rates ranging between 400 and 500 beats per minute. All measurements were averaged across 10 consecutive cardiac cycles.

### Masson Staining

The ischemic or bordering scar region of the left ventricle myocardium were isolated, fixed in 4% paraformaldehyde, and embedded in paraffin. The paraffin sections were sliced at 5 μm and stained with hematoxylin staining solution for 3 min following deparaffinization. Next, the sections were stained with Masson Ponceau acid fuchsin solution for 5–10 min, followed by differentiation with 1% phosphomolybdic acid aqueous solution for 3 min, and stained with aniline blue solution for 5 min. Lastly, the sections were blocked with neutral gum and observed under the microscope (Nikon TE2000-U microscope, Japan). The area of myocardial fibrosis was quantified using Image J (v1.8.0, National Institutes of Health, United States). Myocardial tissues were stained with red; collagenous fibers were presented in blue. Vasculature and scar regions with a high abundance of collagen were excluded from quantification.

### Cardiac Fibroblast Isolation, Culture, and Cell Transfection

Cardiac fibroblasts were obtained by digesting the ventricles of 1- to 2-day-old Sprague–Dawley rats with 0.08% collagenase II (Sigma, United States) and 0.1% trypsin (Beyotime, Shanghai) as previously reported (Tao et al., 2014) and cultured in Dulbecco’s modified Eagle’s medium (DMEM) (high glucose, Gibco) containing 10% fetal bovine serum (FBS) (Gibco, Gaithersburg, United States), 100 U/ml of penicillin, and 100 μg/ml of streptomycin (Beyotime, Shanghai) in 5% CO_2_ at 37°C. The CFs were passaged upon 80–90% confluency. Fibroblasts were only passaged once before conducting further experiments.

Notch3-specific small interfering ribonucleic acid (si notch3) and scrambled siRNA (sc notch3) were synthesized by Genepharm Biotech (Shanghai, China). The siRNA sequences were as follows:

siRNA1: 5′-GCAUCUGCCAUGGAGGAUATT-3′;
siRNA2: 5′-CCUGCAACCCGGUUUAUAATT-3′;
siRNA3: 5′-CCGUGUGGCCUCUUUCUAUTT-3′; and
scramble siRNA: 5′-UUCUCCGAACGUGUCACGUTT-3′.

The N3ICD cDNA was cloned into an expression vector (GV314) and coupled to a Flag tag. Recombinant expression of the pFlag-N3ICD plasmid was confirmed by DNA sequencing (Genechem, Shanghai China). When the CFs reached 60% confluency, si notch3, sc notch3, notch3 overexpression plasmid (ov-N3ICD), or the empty vector plasmid (vector) was transfected into CFs using the Lipofectamine 3000 transfection reagent (Invitrogen). The transfection efficiency was quantified using RT-qPCR and western blotting 48 h after transfection.

To investigate the role of Hif1α and the RhoA/ROCK signaling pathway in the regulation of notch3-dependent effects on CF activity, 2-ME (an inhibitor of Hif1α, MedChemExpress, United States, 10 μmol), DMOG (an inhibitor of Hif-1α prolyl hydroxylase, MedChemExpress, United States, 100 μmol), and Y-27632 (an inhibitor of the RhoA/ROCK pathway, MedChemExpress, United States, 30 μmol) were applied with a 2-h preincubation prior to transfection.

### Real-Time qPCR

Total RNA from CFs and myocardial tissue was extracted using TRIzol (Takara, Japan). Reverse transcription was carried out with the PrimeScript RT reagent Kit with gDNA Eraser (Takara, Japan). The qPCR primers were obtained from Invitrogen (Carlsbad, CA, United States). The primer sequences were as follows:

Notch3 forward primer: 5′-GCACGAACTGACCGAA CTGG-3′;
Notch3 reverse primer: 5′-TGATGAGAATCT GGAAGACACCC-3′;
GAPDH forward primer: 5′-AAGTTCAACGGC ACAGTCAAGG-3′; and
GAPDH reverse primer: 5′-ACGCCAGTAGACT CCACGACAT-3′.

Notch3 gene expression was quantified by PCR Amplification Kit (Takara, Japan). Briefly, the PCR included the following steps: denaturation of cDNA at 95°C for 30 s, followed by 40 cycles of 95°C for 5 s (denaturation) and 60°C for 30 s (annealing and elongation). GAPDH was used as a loading reference. Differential expression values were calculated using the ΔΔCT method.

### Western Blot Analysis

Protein was extracted from isolated CFs and ventricular tissue using radioimmunoprecipitation assay (RIPA) buffer (Beyotime, China) containing protease and a phosphatase inhibitor cocktail (MCE). To separate cytosolic and membrane fractions, we used a membrane and cytosol protein extraction kit (Beyotime, Haimen, China) according to the manufacturer”s instructions. Protein concentrations were quantified with the bicinchoninic acid (BCA) assay (Beyotime, China). Proteins were separated on an 8–12% sodium dodecyl sulfate–polyacrylamide gel electrophoresis (SDS-PAGE) and transferred to polyvinylidene difluoride (PVDF) membranes (Roche Applied Science, Germany). After being blocked with 5% milk for 1 h, the membranes were incubated with primary antibodies at 4°C overnight. We used the following antibodies: notch3 (1:1,000), α-SMA (1:2,000), total caspase3 (1:1,000), GAPDH (1:1,000), Pan cadherin (1:1,000), and β-actin (1:1,000), all from Proteintech (Rosemont, United States); Col I (1:1,000), Hif1α (1:1,000), Col III (1:1,000), and Bcl2 (1:1,000) from GeneTex (Irvine, CA, United States); and RhoA (1:1,000), ROCK1 (1:1,000), and ROCK2 (1:1,000) from Abcam (Cambridge, United States). The membranes were washed with TBST three times and then incubated with horseradish peroxidase (HRP)-conjugated secondary antibodies (Proteintech, Rosemont, United States) at room temperature for 1 h. Images were acquired with the ChemiDoc Imager (Bio-Rad, Hercules, CA, United States).

### Cell Counting Kit-8 Assay

Cell viability was evaluated with Cell Counting Kit-8 (CCK8; MedChemExpress, United States) according to the manufacturer’s instructions. Briefly, CFs were seeded in 96-well culture plates at a density of 5 × 10^3^ cells/well and cultured at 5% CO_2_ at 37°C. After transfection for 48 h, we added 10 μl of CCK8 solution, incubated at 37°C for 1–4 h, and then detected optical density of each well at 450 nm with a microplate reader (Molecular Devices).

### EdU Proliferation Assay

Cardiac fibroblast proliferation was evaluated using an EdU cell proliferation assay kit (Ribobil^TM^, China). The CFs were seeded in 24-well plate, transfected with si notch3 or ov-N3ICD plasmid for 48 h, and incubated with 10 μm EdU for 24 h. We then fixed the cells with 4% paraformaldehyde, permeabilized them with 0.5% Triton-X 100 in phosphate-buffered saline (PBS), stained with EdU, and then counterstained with DAPI (Boster, China). Images were acquired using a Nikon TE2000-U microscope (Tokyo, Japan). The percentage of EdU-positive cells was calculated from six random fields over three wells.

### Immunofluorescence

After transfection with si notch3 or ov-N3ICD plasmid for 12 h, CFs were fixed with 4% paraformaldehyde for 20 min, incubated with 0.1% Triton-X 100 in PBS for 15 min, blocked in 10% goat serum for 30 min, and then incubated with an anti-Hif1α primary antibody (GeneTex, United States, 1:200) overnight at 4°C. After three PBS washing steps, CFs were incubated with DyLight 594-conjugated goat anti-rabbit IgG (red) (EarthOx, Millbrae, United States) at room temperature for 1 h protected from light. The nuclei were counterstained using DAPI (Boster, China) for 5 min. We acquired confocal images at 600 × magnification on a LEICA TCS SP2 confocal microscope. Fluorescence intensities were quantified using Image J (v1.8.0, National Institutes of Health, United States).

### Flow Cytometry

After transfection for 48 h, CFs were digested with 0.25% trypsin and washed three times with PBS. CFs were resuspended in 500 μl of PBS, labeled with Annexin V-APC and propidium iodide (PI), and incubated for 15 min in the dark. Apoptotic cells were detected by flow cytometry sorting of Annexin V and PI double-stained cells (FACS Vantage SE, BD, United States). The apoptotic index was calculated as follows: (number of apoptotic cells/total number of cells tested) × 100%.

### Statistical Analyses

We employed GraphPad Prism 5.0 (GraphPad Software Inc., San Diego, CA, United States) for data analysis. Continuous variables were presented as the mean ± SD. Statistical comparisons were performed by Student’s *t*-test or one-way analysis of variance (ANOVA). All experiments were repeated at least three times. Statistical significance was defined as *P* < 0.05.

## Results

### Notch3 Inhibits the Proliferation of Cardiac Fibroblasts

**FIGURE 1:**
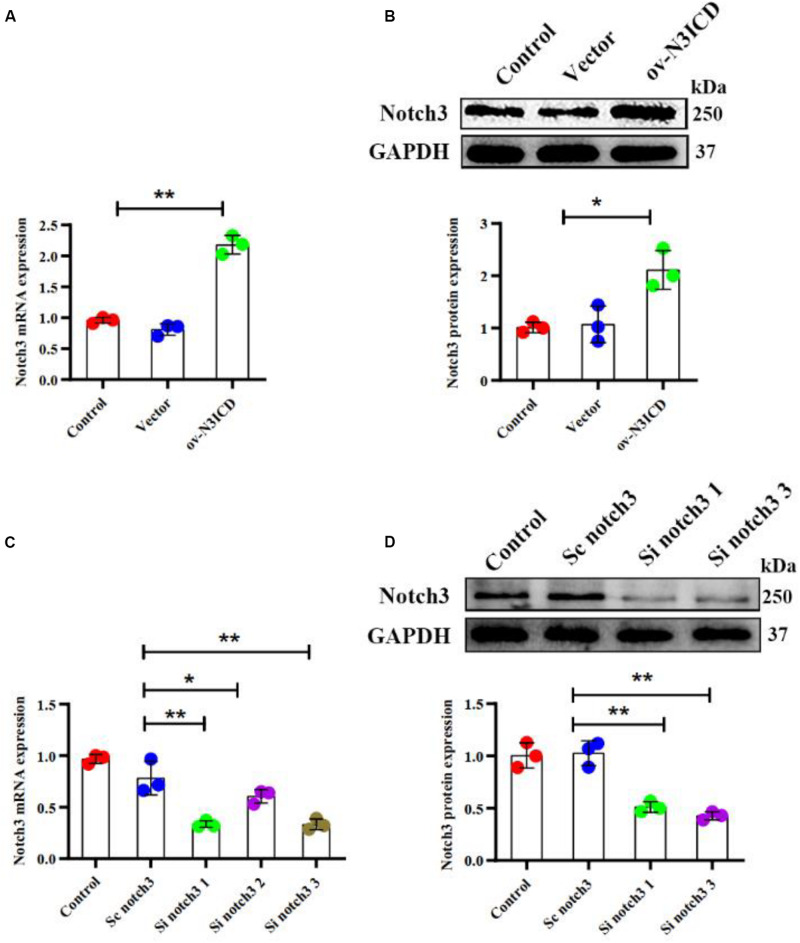
Notch3 was overexpressed and knocked down successfully in cardiac fibroblasts (CFs). **(A,B)** Rat CFs were transfected with ov-N3ICD plasmid or vector. Notch3 expression was detected by RT-qPCR and western blotting analysis (*n* = 3). **(C)** CFs were treated with small interfering RNA constructs (sc notch3, si notch3 1, si notch3 2, or si notch3 3), and mRNA expression of notch3 was quantified by RT-qPCR (*n* = 3). **(D)** Western blot analysis of notch3 expression after notch3 knockdown (*n* = 3). Values represent the mean ± SD. *P < 0.05, **P < 0.01.

To examine the effect of notch3 on CF proliferation, we transfected cells with either overexpression plasmid (ov-N3ICD) or siRNA duplexes (si notch3). After the transfection of the ov-N3ICD plasmid, the mRNA and protein expression levels of notch3 were significantly higher than in the control vector group (Figures 1A,B). For silencing notch3, we constructed three siRNA duplexes (si notch3 1, si notch3 2, and si notch3 3). RT-qPCR confirmed that both si notch3 1 and si notch3 3 effectively knocked down notch3 (Figure 1C). Similarly, western blotting analysis showed that both si notch3 1 and si notch3 3 effectively reduced protein expression of notch3 (Figure 1D). Therefore, we used these two constructs for the following experiments.

**FIGURE 2:**
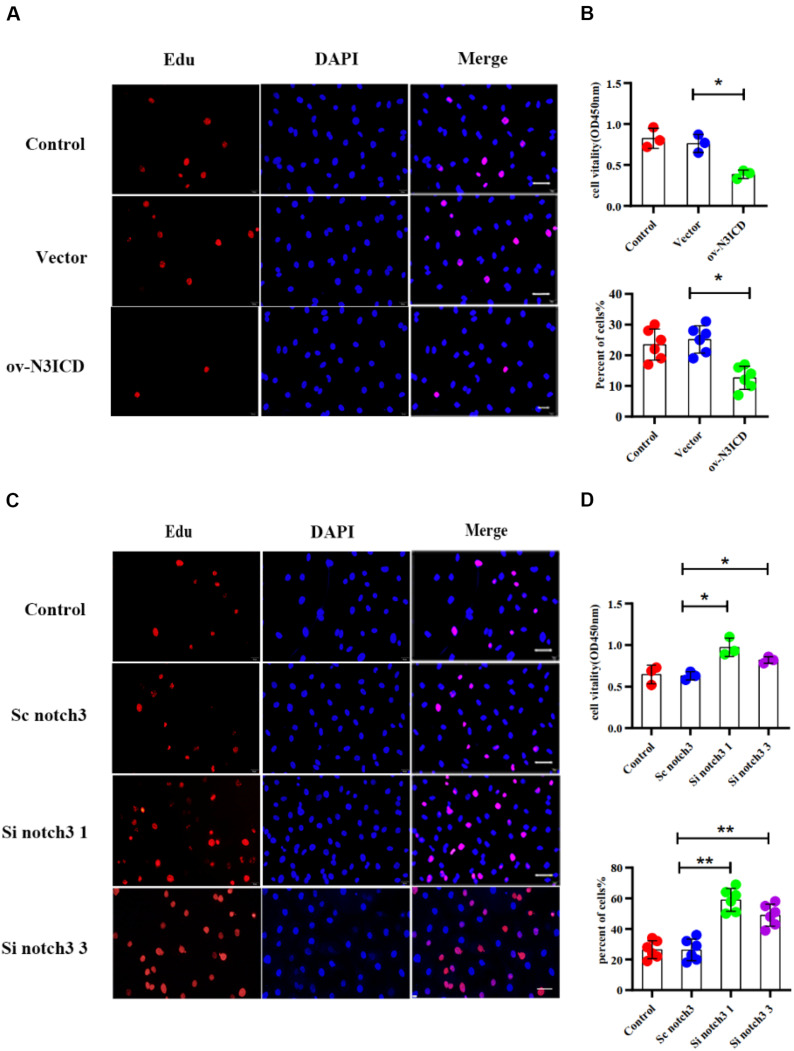
Notch3 inhibits the proliferation of rat cardiac fibroblasts (CFs). **(A,C)** We used the EdU assay to measure CF proliferation after notch3 overexpression or knockdown (*n* = 6). Scale bars = 200 μm. **(B,D)** We next used the Cell Counting Kit-8 (CCK8) assay to determine CF proliferation (above, *n* = 3). Quantification of the CF proliferation determined by EdU assay (below, *n* = 6) and CCK8 assay (above, *n* = 3) in different groups, as indicated. Values represent the mean ± SD. *P < 0.05, **P < 0.01.

To measure the proliferative capacity of CFs after notch3 overexpression or silencing, we carried out EdU and CCK8 staining assays. As shown in Figures 2A,B, CFs in the ov-N3ICD group exhibited a significantly lower proliferation rate than in the vector control group. Conversely, we found that CFs had a higher proliferation rate after siRNA notch3 knockdown than CFs in the scrambled notch3 control group (Figures 2C,D). Therefore, our experiments suggested that notch3 has an inhibitory effect on CF proliferation.

### Notch3 Promotes Cardiac Fibroblast Apoptosis

**FIGURE 3:**
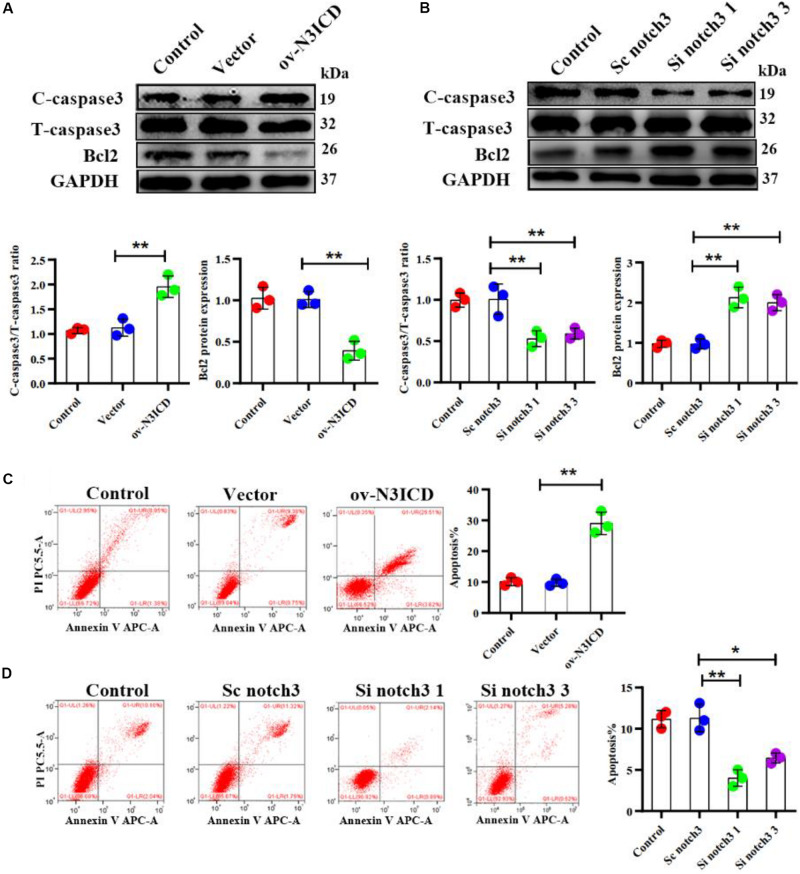
Notch3 promotes the apoptosis of rat cardiac fibroblasts (CFs). **(A,B)** Representative western blot and quantitative data of cleaved caspase3, total caspase3, and Bcl2 after notch3 overexpression or knockdown (*n* = 3). **(C,D)** The CF apoptotic rate after notch3 overexpression or knockdown, detected by flow cytometry. Q1-UR and Q1-LR were used to analyze the change of apoptotic rate in different experimental groups (*n* = 3). Values represent the mean ± SD. *P < 0.05, **P < 0.01. C-caspase3, cleaved caspase3; T-caspase3, total caspase3.

The caspase family plays an important role in the execution of cellular apoptosis. Caspase3, in particular cleaved caspase 3—the active form of caspase3—is widely considered as an apoptotic marker (Zheng et al., 1998; Langford et al., 2011). It is well known that Bcl2 protects many cell lines from apoptosis (Lessene et al., 2008). To further detect anti-apoptotic proteins, we also measured Bcl2 expression. Western blot analysis showed that notch3 overexpression resulted in a significant increase in the ratio of cleaved caspase3 to total caspase3 as well as a significantly lower Bcl2 level as compared with controls (Figure 3A). In contrast, notch3 knockdown exerted an opposite effect on the cleaved caspase3 to total caspase3 ratio and expression levels of Bcl2 (Figure 3B).

Next, we determined the CF apoptosis by flow cytometry with Annexin V and PI double staining. In line with our previous experiments, we found that notch3 overexpression significantly elevated CF apoptosis (Figure 3C), whereas knockdown of notch3 inhibited it (Figure 3D). In summary, these findings suggest that notch3 may promote apoptosis of CFs by increasing the ratio of cleaved caspase3 to total caspase3 and decreasing Bcl2 expression levels.

### Notch3 Inhibits Cardiac Fibroblast to Myofibroblast Transition and Extracellular Matrix Production

**FIGURE 4:**
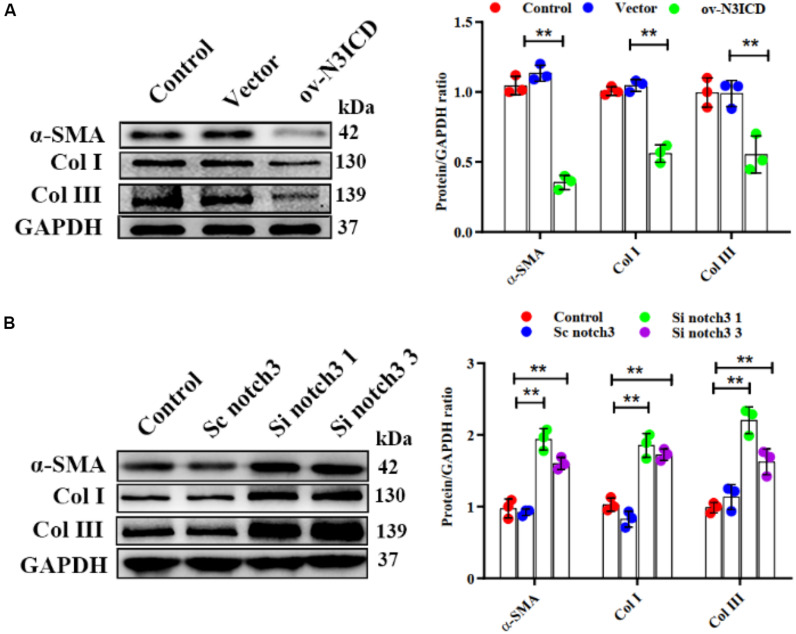
Notch3 inhibits cardiac fibroblast to myofibroblast transition. **(A,B)** Western blots measuring α-SMA, Col I, and Col III after notch3 overexpression or knockdown. The quantification of the protein bands is shown on the right (*n* = 3). Values represent the mean ± SD. *P < 0.05, **P < 0.01.

Previous studies have demonstrated that fibroblast to myofibroblast transition (FMT) and ECM production (Col I and Col III) are key steps in cardiac fibrosis. We therefore quantified α-SMA (a biomarker of myofibroblasts), Col I, and Col III protein levels by western blot. The expression of α-SMA, Col I, and Col III was lower in ov-N3ICD-transfected CFs than in the vector control group (Figure 4A). On the other hand, protein expression of α-SMA, Col I, and Col III increased significantly after notch3 downregulation (Figure 4B). Taken together, these results suggested that notch3 inhibits both the differentiation of CFs into myofibroblasts and ECM production.

### Notch3 Alters the Expression of Hif1α and RhoA/ROCK in Cardiac Fibroblasts

**FIGURE 5:**
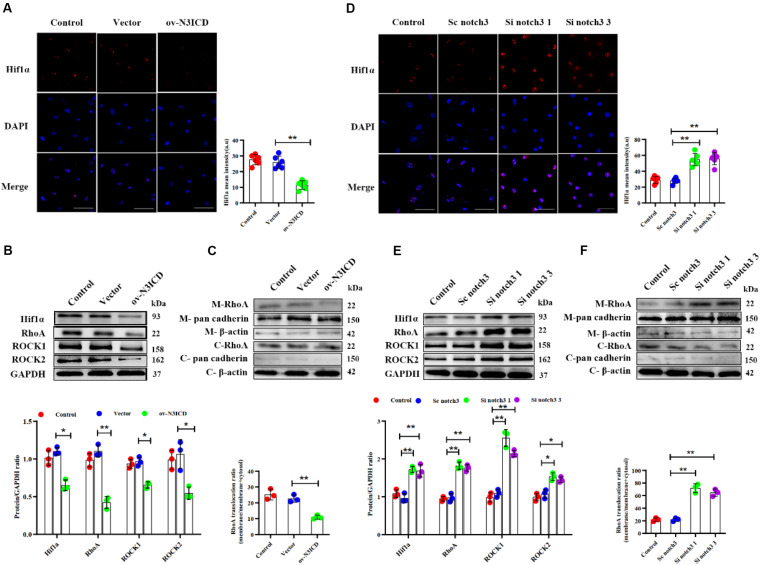
The effects of notch3 on Hif1α, RhoA, ROCK1, and ROCK2 in rat cardiac fibroblasts (CFs) with different treatments. **(A)** Representative immunofluorescence images and quantitative data of Hif1α (red) in neonatal rat cardiac fibroblasts after notch3 overexpression (*n* = 6). Nuclei were detected with DAPI (blue). Scale bars = 200 μm. **(B)** The protein expression of Hif1α, RhoA, ROCK1, and ROCK2 was determined by western blot after notch3 overexpression (*n* = 3). **(C)** Western blots of RhoA in the membrane and cytosol after notch3 overexpression (*n* = 3). **(D)** Hif1α (red) in neonatal rat cardiac fibroblasts after notch3 knockdown was detected by immunofluorescence and statistical analysis of mean intensity (*n* = 6). Nuclei were detected with DAPI (blue). Scale bars = 200 μm. (E) Hif1α, RhoA, ROCK1, and ROCK2 were detected by western blot after notch3 silencing (*n* = 3). **(F)** Western blots of RhoA and cadherins in the membrane and cytosol after notch3 silencing (*n* = 3). Values represent the mean ± SD. *P < 0.05, **P < 0.01. M-RhoA, membrane RhoA; C-RhoA, cytosolic RhoA; M-pan cadherin, membrane pan cadherin; C-pan cadherin, cytosolic pan cadherin; M-β-actin, membrane β-actin; C-β-actin, cytosolic β-actin.

To investigate the effects of notch3 on Hif1α, we quantified Hif1α levels after notch3 overexpression or knockdown, respectively. Immunofluorescence of CFs showed that the nuclear expression of Hif1α was significantly lower after notch3 overexpression (Figure 5A), whereas notch3 knockdown induced Hif1α expression in the nucleus (Figure 5D). The western blotting analysis demonstrated that notch3 overexpression reduced the expression of Hif1α, RhoA, ROCK1, and ROCK2 (Figure 5B). To further evaluate the function of the RhoA/ROCK signaling pathway in CFs, we also measured the activity levels of RhoA. Previous studies suggested that membrane-associated RhoA GTP is the active form of RhoA (Somlyo and Somlyo, 2000) and showed that RhoA activity is correlated with membrane-associated RhoA protein levels (Tamma et al., 2003). Western blot analysis showed that pan cadherin (membrane marker) was strongly expressed whereas β-actin was extremely low in the membrane fractions. However, β-actin was present at a very high concentration, whereas pan cadherin was hardly detected in the cytosolic fractions. These results suggest that the membrane fractions and the cytosolic fractions were separated adequately. We also found that notch3 overexpression reduced the ratio of membrane-associated RhoA to total RhoA (Figure 5C). Conversely, notch3 knockdown changed the expression levels of Hif1α, RhoA, ROCK1, ROCK2, and membrane-bound RhoA (Figures 5E,F).

### Notch3 Regulates Cardiac Fibroblast Proliferation, Apoptosis, and Fibroblast to Myofibroblast Transition via the RhoA/ROCK/Hif1α Pathway

**FIGURE 6:**
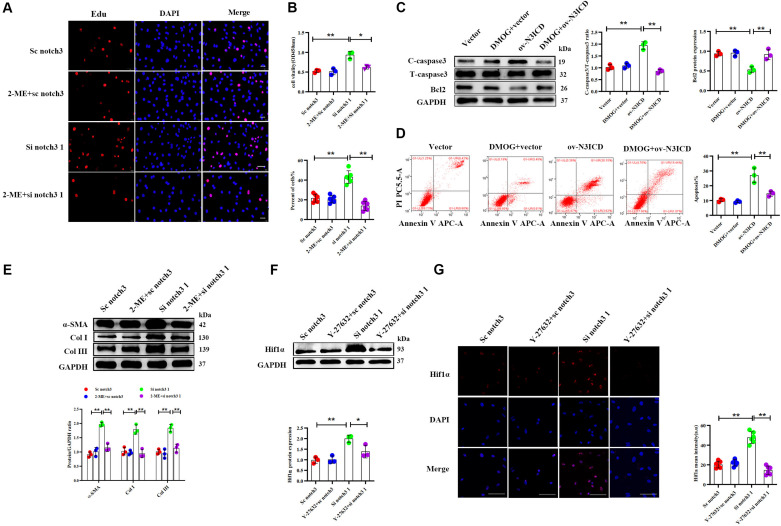
Notch3 regulates cardiac fibroblast (CF) function via the RhoA/ROCK/Hif1α pathway. **(A)** CFs were pretreated with 2-ME for 2 h before notch3 knockdown. CF proliferation was quantified using the EdU assay (*n* = 6). Scale bars = 200 μm. **(B)** The Cell Counting Kit-8 (CCK8) assay was also used to determine CF proliferation (above, *n* = 3). Quantification of CF proliferation determined by EdU assay (below, *n* = 6). **(C)** Western blot analysis of cleaved caspase-3, total caspase3, and Bcl2 in different groups. The quantification of the protein bands is shown on the right (*n* = 3). **(D)** CFs were pretreated with DMOG for 2 h before notch3 overexpression. Flow cytometry quantified the percentage of apoptosis in each group (*n* = 3). **(E)** Western blot analysis of α-SMA, Col I, and Col III in CFs pretreated with 2-ME for 2 h before notch3 knockdown. The quantification of the protein bands is shown below (*n* = 3). **(F)** Representative Western blot and quantification of Hif1α in CFs pretreated with Y-27632 for 2 h before notch3 knockdown (*n* = 3). **(G)** Representative immunofluorescence images and quantification of Hif1α (red) in CFs pretreated with Y-27632 for 2 h before notch3 knockdown (*n* = 6). Nuclei were detected with DAPI (blue). Scale bars = 200 μm. Values represent the mean ± SD. *P < 0.05, **P < 0.01.

Among the two effective siRNA duplexes (si notch3 1 and si notch3 3), si notch3 1 exhibited better interference and was therefore used for the following experiment. To clarify the role of Hif1α in regulation of CF proliferation via notch3, CFs were pretreated with Hif1α inhibitor, 2-ME, for 2 h and then transfected with si notch3 1 for 48 h. The EdU assay and CCK8 staining demonstrated that 2-ME reversed the notch3 knockdown-induced positive regulation on CF proliferation (Figures 6A,B) and resulted in attenuated CF proliferation. Similarly, 2-ME pretreatment could effectively weaken the rise of α-SMA, Col I, and Col III caused by notch3 knockdown (Figure 6E).

We next used DMOG, a potent Hif1α prolyl hydroxylase inhibitor and therefore Hif1α activator, to investigate the interaction between Hif1α and notch3 in the regulation of CF apoptosis. The ratio of cleaved caspase 3 to total caspase 3 and Bcl2 levels were restored when CFs were pretreated with DMOG before ov-N3ICD plasmid transfection (Figure 6C). Meanwhile, we had previously shown by flow cytometry that overexpression of notch3 increases apoptosis; this effect was abolished by DMOG pretreatment (Figure 6D).

Therefore, our results suggested that Hif1α is involved in the notch3-dependent regulation of CFs. To further explore the relationship between the RhoA/ROCK pathway and Hif1α, we applied Y-27632, an inhibitor of RhoA/ROCK pathway, to CFs before notch3 overexpression and knockdown. We found that Y-27632 pretreatment was able to abolish the increase in Hif1α protein expression induced by notch3 knockdown (Figures 6F,G). These results indicated that Hif1α might be a downstream molecule of the RhoA/ROCK signaling pathway in CFs.

Overall, our results therefore indicate that the RhoA/ROCK/Hif1α signaling pathway is associated with the proliferation, apoptosis, and FMT of CFs *in vitro*.

### Notch3 Alleviates Cardiac Fibrosis After Myocardial Infarction via the RhoA/ROCK/Hif1α Pathway

**FIGURE 7:**
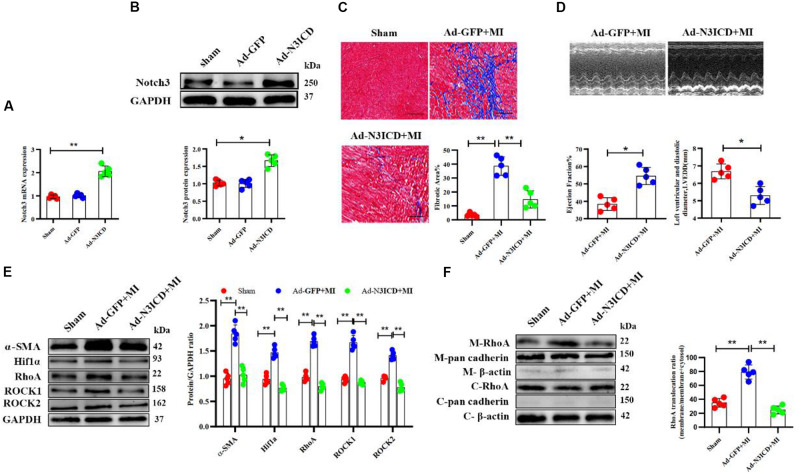
Notch3 alleviates cardiac fibrosis after myocardial infarction via the RhoA/ROCK/Hif1α pathway. **(A,B)** Notch3 overexpression in the myocardium after Ad-N3ICD injection was confirmed by RT-qPCR and western blot (*n* = 5). **(C)** Measurements of cardiac function indices of rats in each group 2 months after myocardial infarction (MI) injury (*n* = 5). **(D)** Masson staining of the rat heart tissues. The positively stained area (blue) represents fibrosis (*n* = 5). **(E)** Western blot analysis and quantitative results of α-SMA, RhoA, ROCK1, ROCK2, and Hif1α (*n* = 5). **(F)** Western blot analysis of membrane-bound RhoA and cadherins in rat hearts from different treatments. Values represent the mean ± SD. *P < 0.05, **P < 0.01. M-RhoA, membrane RhoA; C-RhoA, cytosolic RhoA; M-pan cadherin, membrane pan cadherin; C-pan cadherin, cytosolic pan cadherin; M-β-actin, membrane β-actin; C-β-actin, cytosolic β-actin.

Next, we went on to confirm our findings *in vivo*. We therefore injected an N3ICD-overexpressing adenovirus into myocardium 48 h before LAD ligation to investigate the effects of notch3 on cardiac fibrosis and its underlying mechanism. Both RT-qPCR and western blotting analysis confirmed that notch3 was successfully overexpressed in the myocardium after adenovirus injection (Figures 7A,B). Two months after ligation, echocardiography revealed that Ad-N3ICD + MI animals had a higher LVEF (Figure 7C, 54.60 ± 4.93% vs. 38.40 ± 3.65%, *P* = 0.004) and lower LVEDD (Figure 7C, 6.68 ± 0.43 vs. 5.30 ± 0.51 mm, *P* = 0.017) than the control group (Ad-GFP + MI). Masson staining revealed that myocardial fibrosis area in the Ad-GFP + MI group increased significantly compared with that in the sham group (Figure 7D, 3.54 ± 1.31% vs. 38.58 ± 6.68%, respectively; *P* < 0.001). However, the Ad-N3ICD injection alleviated myocardial fibrosis induced by MI (Figure 7D, 38.58 ± 6.68% vs. 14.62 ± 6.21%, respectively; *P* < 0.001).

Using western blotting, we confirmed that the expression of α-SMA, Hif1α, RhoA, ROCK1, and ROCK2 increased after MI, whereas notch3 overexpression in myocardium can ameliorate these protein expressions induced by MI (Figure 7E). The expression of membrane-bound RhoA increased after MI. However, this effect can be abolished by notch3 overexpression (Figure 7F). Therefore, we conclude that notch3 may alleviate MI-induced cardiac fibrosis via the RhoA/ROCK/Hif1α signaling pathway *in vivo*.

## Discussion

Cardiac fibrosis is a pathological process present in most heart diseases accompanied by loss of myocardium and fibrous tissue replacement, thereby affecting the compliance and function of the ventricle (Kong et al., 2014). CFs predominantly regulate ECM protein synthesis and degradation, and therefore an aberrant and persistent stimulation of CFs has been suggested to be a decisive cause of cardiac fibrosis (Krenning et al., 2010; Travers et al., 2016).

### Notch in Cardiac Fibroblast Proliferation, Apoptosis, and Fibroblast to Myofibroblast Transition

Several studies have confirmed that the notch signaling pathway regulates organ development and fibrosis. However, the exact functions of notch receptors depend on different cellular and physiological pathological conditions. Accumulated evidence shows that notch1 is a protective factor during myocardial fibrosis (Zhou et al., 2015), whereas few researches were reported on notch2, notch3, and notch4. Luxan et al. (2013) reported that inactivation of the notch pathway causes left ventricular non-compaction cardiomyopathy. Urbanek et al. (2010) found that inhibition of notch1-dependent cardiomyogenesis results in cardiomyopathy of the neonatal heart (Urbanek et al., 2010). In addition, Yu and Song (2014) identified that notch1 inhibits cardiomyocyte apoptosis after myocardial ischemia. Besides, notch3 has been suggested to be a protective factor for prevention of post-MI myocardial fibrosis (Zhang et al., 2016), but the underlying molecular mechanism remained unclear. Owing to the crucial role of CFs in myocardial fibrosis, the effects of notch3 on CF activity required further investigation. Therefore, we evaluated CF activity upon both notch3 overexpression and knockdown. Our results revealed that notch3 overexpression inhibits CF proliferation and promotes CF apoptosis. Activated CFs, labeled with the contractile protein α-SMA, secrete large amounts of collagen, which determines myocardial stiffness and compliance. In our study, we found that notch3 overexpression inhibits FMT and decreases protein levels of α-SMA, Col I, and Col III, whereas notch3 downregulation shows inverse effects. Several pathways are involved in ECM maintenance. Here, we investigated one line of notch signaling; however, as ECM deposition is a key regulatory event in fibrosis, other involved molecules, such as matrix metalloproteinases (MMPs), should also be investigated. Future research should therefore address the pathophysiological mechanisms of MMPs in modulating the Notch-dependent fibrosis.

### Notch and the RhoA/ROCK/Hif1α Pathway

Hif1 is ubiquitously expressed in nearly all mammalian cells and is an essential regulator under hypoxia. Hif1 contains two subunits, Hif1α and Hif1β (Imtiyaz and Simon, 2010; Masoud and Li, 2015). Hif1α has been shown to be closely associated with organ fibrosis: Han et al. (2019) suggested Hif1α promotes liver fibrosis by action on the PTEN/p65 pathway in non-alcoholic fatty liver disease. Ichihara et al. (2019) found that Hif1α was elevated in Ang II-induced cardiac fibrosis. In addition, Hif1α has been shown to participate in the activation of the notch signaling pathway in both odontogenic keratocysts (Miranda da Costa et al., 2019) and neurogenesis during acute epilepsy (Li et al., 2018). Moreover, notch signaling can enhance the expression of Hif1α in human adipose-derived mesenchymal stromal cells (Moriyama et al., 2018). Even so, the direct effects of Hif1α on CF activity and its interaction with the notch signaling pathway in CFs remained incompletely understood. Our results showed that Hif1α can be detected in CFs and is downregulated by notch3 overexpression but upregulated by notch3 knockdown.

Hif1α is a central component of the oxygen sensing system. Normoxia provokes ubiquitin–proteasome system, thereby promoting Hif1α degradation (Huang et al., 1998; Kaelin and Ratcliffe, 2008). Therefore, the expression of Hif1α is very low under normoxia and even at an undetectable level. Besides hypoxia, Hif1α can be also induced by inflammatory cytokines, growth factors, and hormones under normoxic conditions (Zhou and Brüne, 2006). McMahon et al. (2006) have demonstrated TGF-β1 enhances Hif1α protein stability by inhibiting PHD2 expression in hepatoma cells. Our present research found that Hif1α could be detected and is regulated by the notch signaling pathway under normoxic conditions. Taken together, these results suggest that Hif1α can be activated by both hypoxia-dependent and hypoxia-independent pathways. To clarify the relationship between Hif1α and notch signaling pathway in a normoxic environment, CFs were pretreated with 2-ME (Hif1α inhibitor) or DMOG (Hif1α enhancer) before transfection with notch3 overexpressing or silencing constructs. We found that 2-ME weakened the positive regulation of notch3 interference on α-SMA, Col I, Col III, and CF proliferation, whereas DMOG abolished the effect of notch3 on CF apoptosis. To our knowledge, our results describe for the first time that notch3 is capable of inhibiting the expression of Hif1α in CFs and regulates CF activity by Hif1α inactivation.

We further investigated how notch3 regulates Hif1α levels in CFs. We found that the RhoA/ROCK signaling pathway potentially acts as a molecular bridge, connecting notch and Hif1α in the regulation of CF activity. The RhoA/ROCK signaling pathway has been shown to be involved in cardiac protection (Lee et al., 2014; Lai et al., 2017), but the effects of the notch on the RhoA/ROCK signaling pathway vary in different pathological processes. In the ischemic liver, the RhoA/ROCK pathway is activated by Notch1 deficiency (Lu et al., 2018). On the other hand, notch activates the RhoA/ROCK pathway in endothelial barrier dysfunction (Venkatesh et al., 2011). Takata et al. (2008) reported that a Rho-kinase inhibitor, fasudil, prevents Hif1α expression under hypoxia in endothelial cells. Our results suggest that notch3 regulates CF activity via suppression of the RhoA/ROCK pathway. We used Y-27632, RhoA/ROCK pathway inhibitor, to confirm the relationship between the RhoA/ROCK pathway and Hif1α in CFs, and we found that inhibition of the RhoA/ROCK pathway attenuated a notch3-knockdown-induced increase in Hif1α levels. These results provide evidence for a role of notch3 in modulating CF activity by negative regulation of the RhoA/ROCK/Hif1α axis *in vitro*.

Previous studies have confirmed that notch overexpression in rat heart is capable of preventing cardiac fibrosis after MI (Rodriguez et al., 2019; Zhou et al., 2019). Similarly, we confirmed our findings with an *in vivo* rat MI model. Consistent with previous studies, Masson staining and α-SMA expression levels showed that notch3 overexpression could alleviate MI-induced myocardial fibrosis. We furthermore found that notch3 overexpression inhibited the protein expression of RhoA, ROCK1, and ROCK2. Therefore, it is plausible that notch3 interacts with the RhoA/ROCK/Hif1α pathway to inhibit pathologic CF activity and further prevent MI-related cardiac fibrosis in the rat hearts.

A key limitation of our research is that we did not measure the local oxygen concentration in CFs and were therefore unable to determine whether CFs experience intrinsic hypoxia and whether notch3 affects intracellular oxygen balance or not. So far, our results only confirm that notch3 regulates CF activity by inhibiting the RhoA/ROCK/Hif1α axis in a normoxic environment.

## Conclusion

Our study demonstrates, for the first time, that notch3 inhibits both CF proliferation and cardiac FMT and promotes apoptosis via inhibition of the RhoA/ROCK/Hif1α signaling pathway to ultimately attenuate MI-induced myocardial fibrosis. These findings are significant for the further understanding of cardiac fibrosis pathogenesis and provide new therapeutic avenues for future treatment of cardiac fibrosis.

## Ethics Statement

The animal study was reviewed and approved by the Institutional Ethics Committee of Chongqing Medical University.

## Author Contributions

All authors listed have made a substantial, direct and intellectual contribution to the work, and approved it for publication.

## Conflict of Interest

The authors declare that the research was conducted in the absence of any commercial or financial relationships that could be construed as a potential conflict of interest.
